# Restrictive IgG antibody response against mutated citrullinated vimentin predicts response to rituximab in patients with rheumatoid arthritis

**DOI:** 10.1186/s13075-015-0717-z

**Published:** 2015-08-13

**Authors:** Luisa Lindenberg, Lydia Spengler, Holger Bang, Thomas Dorner, Aleksej L Maslyanskiy, Sergey V Lapin, Elena I Ilivanova, Lorena Martinez-Gamboa, Hans Bastian, Esther Wittenborn, Karl Egerer, Gerd-R Burmester, Eugen Feist

**Affiliations:** Department of Rheumatology and Clinical Immunology, Charité-University Medicine, Chariteplatz 1, 10117 Berlin, Germany; Orgentec Diagnostika GmbH, Carl-Zeiss-St. 49, Mainz, 55129 Germany; Immanuel Hospital, Clinic of Rheumatology, Lindenberger Weg 19, Berlin-Buch, 13125 Germany; Roche Pharma, Rheumatology, Emil-Barell-St 1, Grenzach-Wyhlen, 79639 Germany; Federal Almazov, Medical Research Centre, Akkuratova street 2, St. Petersburg, 197341 Russia; St. Petersburg State Pavlov Medical University, Center for Molecular Medicine, ul. Lva Tolstogo 6-8, St. Petersburg, 197022 Russia; Rheumatology Department, Leningrad Regional Clinical Hospital, Lunacharskogo pr. 49, St. Petersburg, 194291 Russia; Labor-Berlin GmbH, Sylter St 2, Berlin, 13353 Germany

## Abstract

**Introduction:**

Antibodies against mutated citrullinated vimentin (AMCV) represent a useful diagnostic marker with correlation to disease activity in patients with rheumatoid arthritis (RA). Since seropositivity for citrullinated autoantibodies was predictive for response to B-cell depleting therapy (BCDT) with rituximab (RTX), we investigated whether differences in antibody fine reactivity and immunoglobulin (Ig) isotype kinetics among AMCV-positive patients could provide additional information about outcome.

**Methods:**

A total of 50 AMCV IgG-positive RA patients (RTX responders (RRs) n = 37 and non-responders (NRRs) n = 13) were analyzed for reactivity against MCV epitopes and co-existent AMCV isotypes IgM and IgA. Antibody titers were determined by enzyme-linked immunosorbent assay at baseline and 24 weeks after the first cycle of RTX, and compared to kinetics of rheumatoid factor (RF) and antibodies against cyclic citrullinated peptide (ACCP).

**Results:**

Recognized MCV epitopes by AMCV IgG of RRs and NRRs showed similar baseline patterns, with reducing reactivity in RRs and unchanged or even expanding reactivity in NRRs upon RTX treatment. At baseline, RRs were more frequently negative for AMCV subtypes, especially for IgA (68 %), compared to NRRs (31 %). Being AMCV IgA-negative at baseline indicated a good treatment response to RTX (negative predictive value = 0.86). Co-existence of AMCV IgA and IgG with stable titers upon treatment were associated with poorer responses to RTX. Furthermore, reductions of AMCV IgA levels upon RTX correlated with the improvement of 28-joint Disease Activity Score (DAS28). In comparison, subtypes of RF and ACCP were not of additional value for prediction of RTX response.

**Conclusions:**

Restrictive IgG seropositivity against MCV with treatment-associated decline in fine reactivity and titers was predictive for response to RTX. Double-positivity for AMCV IgG and IgA was associated with failure to respond to BCDT, suggesting a pathogenetic and less sensitive IgA-producing B-cell subset in NRRs.

**Electronic supplementary material:**

The online version of this article (doi:10.1186/s13075-015-0717-z) contains supplementary material, which is available to authorized users.

## Introduction

Rheumatoid arthritis (RA) is one of the most common systemic autoimmune diseases worldwide, characterized by chronic and erosive arthritis, as well as by an increased mortality, mainly due to infections, cardiovascular events and malignant lymphoma [1]. Early diagnosis and treatment with synthetic and biological disease-modifying anti-rheumatic drugs (DMARDs) is crucial for remission of RA [2]. Since its authorization in 2006, biological treatment with rituximab (RTX) was approved in cases of inadequate response to tumor necrosis factor-alpha inhibitors (TNFi) [3]. Due to its beneficial safety profile and cost-efficacy, RTX has been currently recommended as the first-line biologic treatment in many countries worldwide [4]. Nevertheless, up to one third of RA patients still fail to respond to biologics, including RTX, leading to an individual as well as medical and economic burden [5]. Therefore, to facilitate a more personalized medicine, predictive biomarkers for response are urgently needed.

Response to a B-cell targeted therapy with RTX is usually evaluated by clinical and laboratory signs (cellular and humoral parameters) [6–8]. Due to the mode of action of RTX, follow-up investigations on cellular subsets and humoral factors were of special interest reflecting clinical response to RTX [9]. RA-associated autoantibodies, like rheumatoid factor (RF) and anti-citrullinated protein antibodies (ACPAs), were shown to be of predictive value for response to RTX [6, 10, 11]. In this context, RF showed treatment-related reductions, whereas antibodies against cyclic citrullinated peptide (ACCP) remained rather stable over the course of treatment [12, 13]. Of note, ACPA-seropositivity includes heterogeneous fine specificities against diverse citrullinated proteins [14]. Antibodies against MCV (AMCV) were shown to be highly sensitive and to correlate with disease activity of RA, probably due to the synovial appearance of the antigen during inflammation [15–18]. In particular, a concomitant presence of immunoglobulin (Ig) A with IgG AMCV was associated with a severe disease course, suggesting predictive properties of AMCV isotypes [19]. AMCV positivity was also postulated to predict (moderate) RTX response, but AMCV kinetics, especially of isotypes under B-cell depletion therapy (BCDT), have not been studied in greater detail so far [6]. Although seropositivity of autoantibodies seems to be a positive predictor for response to BCDT, some seropositive patients respond to biological treatment insufficiently.

An objective of this study was to differentiate subgroups of seropositive patients for response to RTX. For this purpose, we investigated the epitope recognition patterns against MCV and the AMCV isotypes in AMCV IgG-positive patients with RA, in relation to their therapeutic outcome to RTX. The aim was to determine a predictive and monitoring parameter for RTX treatment, and to gain further insights into the differential behavior of humoral autoimmune responses under such targeted therapies.

## Methods

### Patients

Our cohort was comprised of AMCV IgG-seropositive RA patients (n = 50) fulfilling the new ACR/EULAR classification criteria [20], who were recruited from the out-patient clinic of the Department of Rheumatology at the Charité-Universitätsmedizin Berlin, Germany (n = 37), and from the Federal Almazov Heart, Blood and Endocrinology Centre, St Petersburg, Russia (n = 13). Patients’ characteristics are summarized in Additional file 1. All patients were inadequate responders to conventional DMARDs, including methotrexate (MTX). RTX was applied in 30 patients as a second-line biologic after failure to TNFi-treatment. A total of 20 patients were biologically naïve and received RTX as a first-line biologic. Before RTX application, all patients had active diseases despite stable treatment with MTX (10 to 25 mg/week), as defined by 28-joint Disease Activity Score based on erythrocyte sedimentation rate (DAS28-ESR) >3.2. The first cycle of RTX (1,000 mg on days one and 15) was administered after standard premedication, and patients were followed up on for a period of six months. A good EULAR response to RTX treatment was evaluated by DAS28-ESR and defined as an improvement of ≥1.2 from baseline to end of follow-up period [21], identifying 37 (74 %) responders to RTX (RRs) and 13 (26-%) non-responders to RTX (NRRs). RRs showed a higher DAS28 at baseline compared to NRRs (6.23 versus 5.09). After the first RTX cycle, DAS28 was significantly reduced in RRs compared to NRRs (3.30 versus 4.72). However, according to EULAR response criteria [22], only a minority of RRs achieved either remission or low disease activity state at week 24 (see Additional file 1). After subsequent cycles of RTX, a further improvement in DAS28 was observed in RRs (data not shown). The study was approved by the local Ethics Committees at the Charité-Universitätsmedizin Berlin, Germany (vote EA1/193/10 april 26, 2012), and the Federal Almazov Heart, Blood and Endocrinology Centre, St. Petersburg, Russia (vote №124 may 21, 2012), and informed consent was given by all patients prior to serum sampling.

### Methods

Anti-MCV reactivities in RRs and NRRs were investigated by an MCV epitope mapping of AMCV IgG, and by determination of AMCV isotype profiles in both groups at baseline and after 24 weeks of RTX-treatment.

From the patients in our cohort, the antibody reactivities of 34 AMCV IgG-positive RA patients (23 RRs compared with 11 NRRs) were tested against 88 epitopes of MCV using enzyme-linked immunosorbent assay (ELISA). Referring to the MCV sequence, 18-mer peptides with 12 overlapping amino acid residues to the adjacent peptide were generated (with the general structure Biotin-SGSG-PEPTIDE-Amide, JPT Peptide Technologies GmbH, Berlin, Germany). Mutations (from glycerine to citrulline and serine to histidine) and citrullinations (replacement of each arginine by citrulline) were inserted. The resulting peptides were applied in ascending order corresponding to the wells of a microtiter plate (A1-H1 (peptide 1–8), A2-H2 (peptide 9–16), A3-H3 (peptide 17–24), A4-H4 (peptide 25–32), A5-H5 (peptide 33–40), A6-H6 (peptide 41–48), A7-H7 (peptide 49–56), A8-H8 (peptide 57–64), A9-H9 (peptide 65–72), A10-H10 (peptide 73–80), A11-H11 (peptide 81–88)) (Additional file 2). Additional to the 88 peptides, biotinylated recombinant MCV, protein A and rheumatoid factor antigen were used as internal controls in a separate row of the microtiter plate (results not shown). Microtiter plates covering the entire peptide sequence were incubated with each patient’s serum sample in a dilution of 1:100 (Orgentec Diagnostika, Mainz; Germany and Medipan GmbH, Berlin/Dahlewitz, Germany) and 1:101 (Generic Assays GmbH, Berlin/Dahlewitz, Germany) in sample buffer (Phosphate-buffered saline [PBS] + 0.1% Tween-20 [NaCl, 1.37 mM; KCl 27 mM; Na2HPO4 100 mM; KH2PO4 18 mM; 0.1 % Tween; pH 7.2], all chemicals from Sigma-Aldrich Chemie GmbH, Taufkirchen, Germany) for 30 minutes. After a washing step, peroxidase conjugated anti-human IgG was added for 30 minutes. After a second washing step, the enzyme- triggered reaction (Dianova GmbH, Hamburg, Germany) was performed by an incubation with 3,3,5,5′-tetra-methyl benzidine (TMB, Life Technologies GmbH, Darmstadt, Germany) as peroxidase substrate for 15 minutes and terminated by H2SO4 (Sigma-Aldrich Chemie GmbH, Taufkirchen, Germany) stop solution. Visualization and quantification of reaction products was made by absorbance detection at 450/620 nm. The cut-off level was set at 300 mOD, following receiver operating characteristic (ROC) analysis.

Immunoglobulin isotypes IgG, IgM and IgA against MCV were measured before and 24 weeks after the first application of RTX in an expanded cohort of 50 patients. Additionally, antibody subtypes of RF IgG, IgA, IgM and ACCP IgG were determined. Detection of all antibodies and subtypes was performed using ELISA. AMCV IgG, IgA and IgM were detected by ELISA (Orgentec Diagnostika, Mainz, Germany) with a cut-off level of 20 U/ml (ROC curve analysis for AMCV IgA test is shown in Additional file 3). RF IgG, IgM and IgA were determined by ELISA (Generic Assays GmbH, Dahlewitz/Berlin, Germany) with a cut-off level for RF IgG and IgA of 30 IU/ml, and 15 IU/ml for IgM. ACCP IgG (second-generation assay) was measured by ELISA (Medizym® anti-CCP, Medipan GmbH, Dahlewitz/Berlin, Germany) with a cut-off level of 30 U/ml. All tests were performed according to the manufacturers’ instructions.

### Statistics

The comparison of antibody reactivities was performed by non-parametric tests with GraphPad Prism 4 (GraphPad Software, Inc., La Jolla, US). The Wilcoxon signed rank test was applied to compare the autoantibody titer difference from baseline to week 24 for RRs and NRRs. In order to compare the titers of RRs with NRRs for each autoantibody subtype, for differences at baseline and at week 24, we used the Mann–Whitney U test. Furthermore, the frequency distribution of seropositivity of antibody subtypes in RRs and NRRs was tested by the chi-squared and Fisher’s exact test. *P* values lower than 0.05 were considered to be significant. For correlation analysis, Pearson coefficients were calculated.

## Results

### Epitope recognition reactivities to mutated citrullinated vimentin peptides of anti-mutated citrullinated vimentin IgG in responders and non-responders to rituximab treatment

**Fig. 1:**
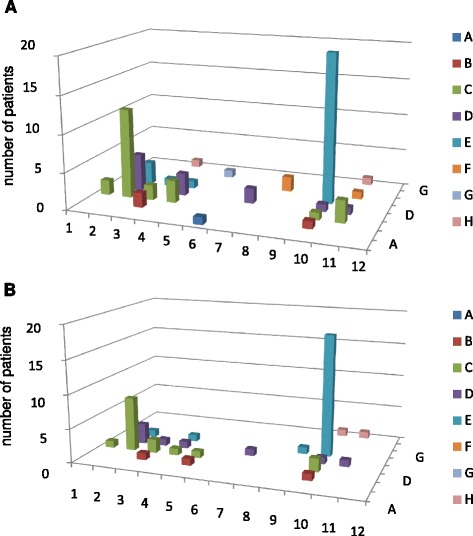
MCV epitope mapping in responders to RTX. The pattern of recognized MCV epitopes by IgG anti-MCV antibodies (AMCV) of RTX responders (n = 23) were reduced from baseline (**a**) to 24 weeks (**b**) after RTX treatment (x axis: wells A to H; y axis: wells 1 to 12; z axis: number of positive patients)

**Fig. 2:**
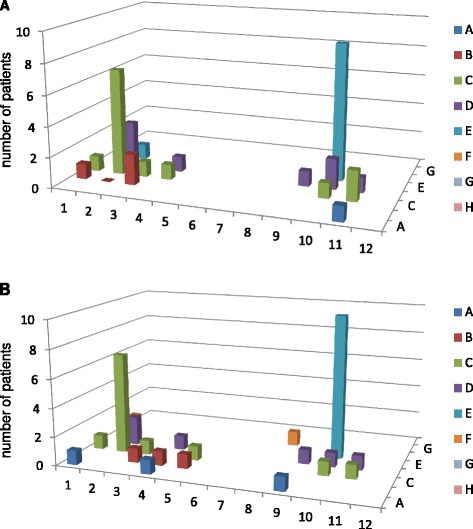
MCV epitope mapping in non-responders to RTX. The pattern of recognized MCV epitopes by IgG anti-MCV antibodies (AMCV) of RTX non-responders (n = 11) remained stable or even expanded from baseline (**a**) to 24 weeks (**b**) after RTX treatment (x axis: wells A to H; y axis: wells 1 to 12; z axis: number of positive patients)

In order to clarify whether AMCV IgG-positive RRs (n = 23) and NRRs (n = 11) to RTX differ in their AMCV reactivities, we performed an epitope mapping using overlapping MCV peptides that were recognized by AMCV IgG of the patients. An illustration of the recognition pattern is shown for RRs (Fig. 1) and NRRs (Fig. 2) as they were targeted on the microtiter plate (x axis: wells A to H; y axis: wells 1 to 12; z axis: number of positive patients). At baseline, only minor differences could be detected between both groups. In NRRs, antibody reactivity against several sequences of the MCV protein with few predominant epitopes (A4, A5, C5 and D5) could be observed. After RTX treatment, differences between RRs and NRRs became more apparent: 69.6 % (16 out of 23) of the RRs and only 27.3 % (three out of 11) of the NRRs showed a reduction of their initial recognition pattern. Moreover, epitope recognition in 45.4 % (five out of 11) of NRRs remained unchanged, and expanded in 27.3 % ( three out of 11).

### Anti-mutated citrullinated vimentin isotype profiles at baseline in responders and non-responders to rituximab

**Fig. 3:**
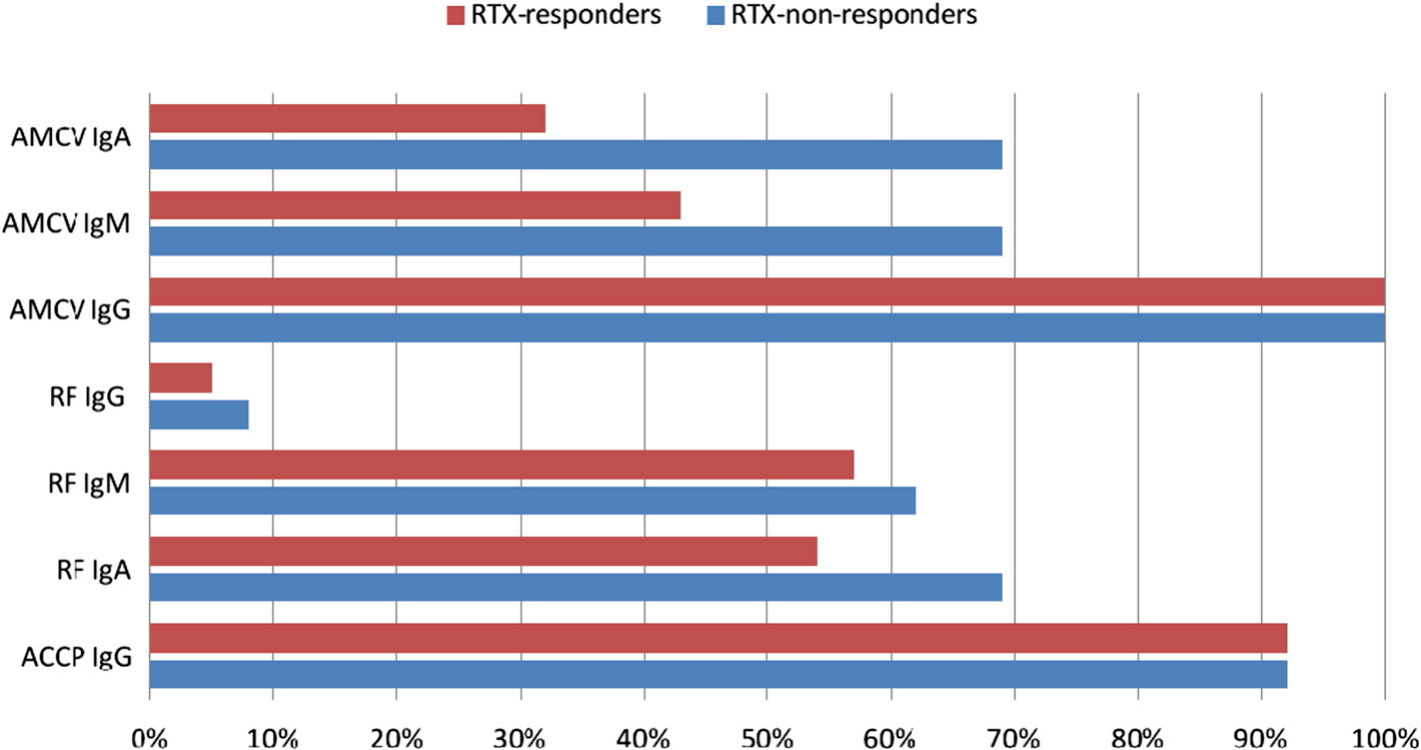
Baseline autoantibody profiles of responders and non-responders to RTX. Non-responders (blue) more frequently showed IgA and IgM antibodies against MCV (AMCV) than responders (red), whereas the antibody distribution of rheumatoid factor (RF) and antibodies against cyclic citrullinated peptide was nearly similar in both groups

In order to clarify whether the response outcome among seropositive patients is associated with Ig isotypes, we analyzed the qualitative distribution of IgM and IgA in AMCV IgG-positive patients and compared them with isotypes of RF and ACCP IgG. At baseline, a lower prevalence of AMCV subtypes was observed in RRs compared to NRRs, with significant differences for AMCV IgA (Table 1 and Fig. 3). In detail, one third (12 out of 37) of the RRs were positive for AMCV IgA titer, whereas 69 % (9/13) of the NRRs were seropositive at baseline (χ^2^*P* = 0.0203). Being AMCV IgA-negative at baseline with an isolated IgG response could indicate a better response to RTX, (negative predictive value = 86.02 %). In return, baseline double-positivity of IgA and IgG was predictive for a poor response to RTX (positive predictive value = 42.86 %).

**Table 1:** Baseline and follow-up characteristics of anti-MCV isotypes for responders compared to non-responders to RTX treatment, respecting baseline seropositivity, mean titers and relative titer changes upon RTX treatment

50 RA patients upon RTX treatment	RRs (n = 37)		NRRs (n = 13)	
	AMCV IgG			
Positive at baseline (n, %)	37	100 %	13	100 %
Negative at baseline (n, %)	0	0 %	0	0 %
Mean baseline titer (v ± SD in U/ml)	769.05	891.04	856.99	982.98
Titer decrease (n, %)	31	84 %	11	85 %
Titer increase (n, %)	6	16 %	1	7.5 %
Mean titer at 24 weeks (v ± SD in U/ml)	390.46	432.06	662.67	834.47
Percentage decline/Wilcoxon *P* value	49.22 %	*P* <0.0001	22.68 %	*P* = 0.02
Mann–Whitney U *P* value (24 weeks)	*P* = 0.288			
Seronormalization (n, %)	4	11 %	1	8 %
Seroconversion (n, %)	0	0 %	0	0 %
	AMCV IgM			
Positive at baseline (n, %)	16	43 %	9	69 %
Negative at baseline (n, %)	21	57 %	4	31 %
Mean baseline titer (v ± SD in U/ml)	39.97	94.43	106.06	147.25
Titer decrease (n, %)	33	89 %	8	62 %
Titer increase (n, %)	4	11 %	5	38 %
Mean titer at 24 weeks (v ± SD in U/ml)	10.41	10.84	39.80	48.62
Percentage decline/Wilcoxon *P* value	73.95 %	*P* <0.0001	62.5 %	*P* = 0.03
Mann–Whitney U *P* value (24 weeks)	*P* = 0.0003			
Seronormalization (n, %)	11	69 %	3	33 %
Seroconversion (n, %)	0	0 %	1	25 %
	AMCV IgA			
Positive at baseline (n, %)	12	32 %	9	69 %
Negative at baseline (n, %)	25	68 %	4	31 %
Mean baseline titer (v ± SD in U/ml)	90.14	240.85	182.51	483.40
Titer decrease (n, %)	32	87 %	9	69 %
Titer increase (n, %)	5	13 %	4	31 %
Mean titer at 24 weeks (v ± SD in U/ml)	29.84	89.06	218.57	513.17
Percentage decline/Wilcoxon *P* value	67 %	*P* <0.0001	19.76 %	*P* = 0.17
Mann–Whitney U *P* value (24 weeks)	*P* = 0.007			
Seronormalization (n, %)	5	42 %	2	22 %
Seroconversion (n, %)	0	0 %	0	0 %

AMCV, antibodies against mutated citrullinated vimentin; n, number of patients; NRRs, non-responders to RTX; RRs, responders to RTX; RTX, rituximab; SD, standard deviation; v, value

Similar but less significant findings were observed for the qualitative difference of AMCV IgM in RRs and NRRs (Table 1). RRs were also less frequently AMCV IgM-positive at baseline compared to NRRs (43 % versus 69 %), but this difference did not reach statistical significance (χ^2^*P* = 0.105). In contrast, IgG, IgM and IgA subclasses of RF, as well as ACCP IgG, showed nearly similar distributions in RRs and NRRs without apparent differences at baseline (Table 2 and Fig. 3).

**Table 2:** Baseline and follow-up characteristics of rheumatoid factor and antibodies against CCP isotypes for responders and non-responders to RTX, respecting baseline seropositivity, mean titers and their relative changes upon RTX treatment

50 RA patients upon RTX treatment	RRs (n = 37)		NRRs (n = 13)	
	RF IgG			
Positive at baseline (n, %)	2	5 %	1	8 %
Negative at baseline (n, %)	35	95 %	12	92 %
Mean baseline titer (v ± SD in IU/ml)	11.99	15.99	13.69	12.30
Titer decrease (n, %)	37	100 %	13	100 %
Titer increase (n, %)	0	0 %	0	0 %
Mean titer at 24 weeks (v ± SD in IU/ml)	3.07	0.97	4.47	3.48
Percentage decline/Wilcoxon *P* value	74.38 %	*P* <0.0001	67.32 %	*P* = 0.002
Mann–Whitney U *P* value (24 weeks)	*P* = 0.224			
Seronormalization (n, %)	2	100 %	1	100 %
Seroconversion (n, %)	0	0 %	0	0 %
	RF IgM			
Positive at baseline (n, %)	21	57 %	8	62 %
Negative at baseline (n, %)	16	43 %	5	38 %
Mean baseline titer (v ± SD in IU/ml)	44.48	69.40	86.22	102.8
Titer decrease (n, %)	36	97 %	11	85 %
Titer increase (n, %)	0	0 %	2	15 %
Mean titer at 24 weeks (v ± SD in IU/ml)	16.54	20.85	58.67	81.14
Percentage decline/Wilcoxon *P* value	62.82 %	*P* <0.0001	31.95 %	*P* = 0.011
Mann–Whitney U *P* value (24 weeks)	*P* = 0.170			
Seronormalization (n, %)	8	38 %	1	13 %
Seroconversion (n, %)	0	0 %	0	0 %
	RF IgA			
Positive at baseline (n, %)	20	54 %	9	69 %
Negative at baseline (n, %)	17	46 %	4	31 %
Mean baseline titer (v ± SD in IU/ml)	102.35	124.7	167.6	144.7
Titer decrease (n, %)	30	81 %	10	77 %
Titer increase (n, %)	5	14 %	2	15 %
Mean titer at 24 weeks (v ± SD in IU/ml)	60.77	94.57	124.0	128.4
Percentage decline/Wilcoxon *P* value	40.62 %	*P* <0.0001	26.05 %	*P* = 0.014
Mann–Whitney U *P* value (24 weeks)	*P* = 0.147			
Seronormalization (n, %)	7	35 %	1	11 %
Seroconversion (n, %)	1	6 %	0	0 %
	ACCP IgG			
Positive at baseline (n, %)	34	92 %	12	92 %
Negative at baseline (n, %)	3	8 %	1	8 %
Mean baseline titer (v ± SD in U/ml)	1125.71	700.0	1044.35	775.6
Titer decrease (n, %)	29	78 %	8	62 %
Titer increase (n, %)	5	14 %	4	31 %
Mean titer at 24 weeks (v ± SD in U/ml)	841.11	667.2	861.55	682.1
Percentage decline/Wilcoxon *P* value	25.28 %	*P* <0.0001	17.50 %	*P* = 0.129
Mann–Whitney U *P* value (24 weeks)	*P* = 0.982			
Seronormalization (n, %)	0	0 %	0	0 %
Seroconversion (n, %)	0	0 %	0	0 %

ACCP, antibodies against cyclic citrullinated peptide; n, number of patients; NRRs, non-responders to RTX; RF, rheumatoid factor; RRs responders to RTX; RTX, rituximab; SD, standard deviation; v, value

Taken together, in our cohort RRs to RTX were characterized by an isolated IgG AMCV response at baseline. In return, co-existent AMCV IgA were able to indicate a poorer response to RTX.

### Anti-mutated citrullinated vimentin isotype titer courses in responders and non-responders upon rituximab treatment

**Fig. 4:**
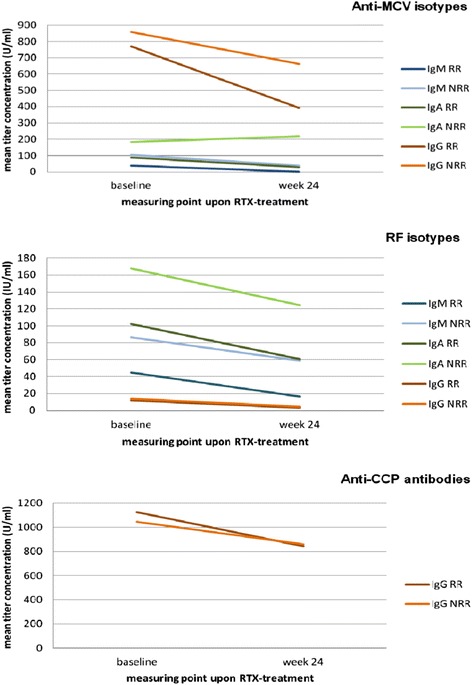
Autoantibody isotype-related mean titer change of responders (RRs) and non-responders (NRRs) upon RTX -treatment. Mean titer course of IgM and especially IgA antibodies against MCV (AMCV) were significantly different in RRs and NRRs to RTX, with higher reductions in RRs after six months. Changes in RF isotypes and antibodies against CCP (ACCP) showed no significant difference between groups

**Fig. 5:**
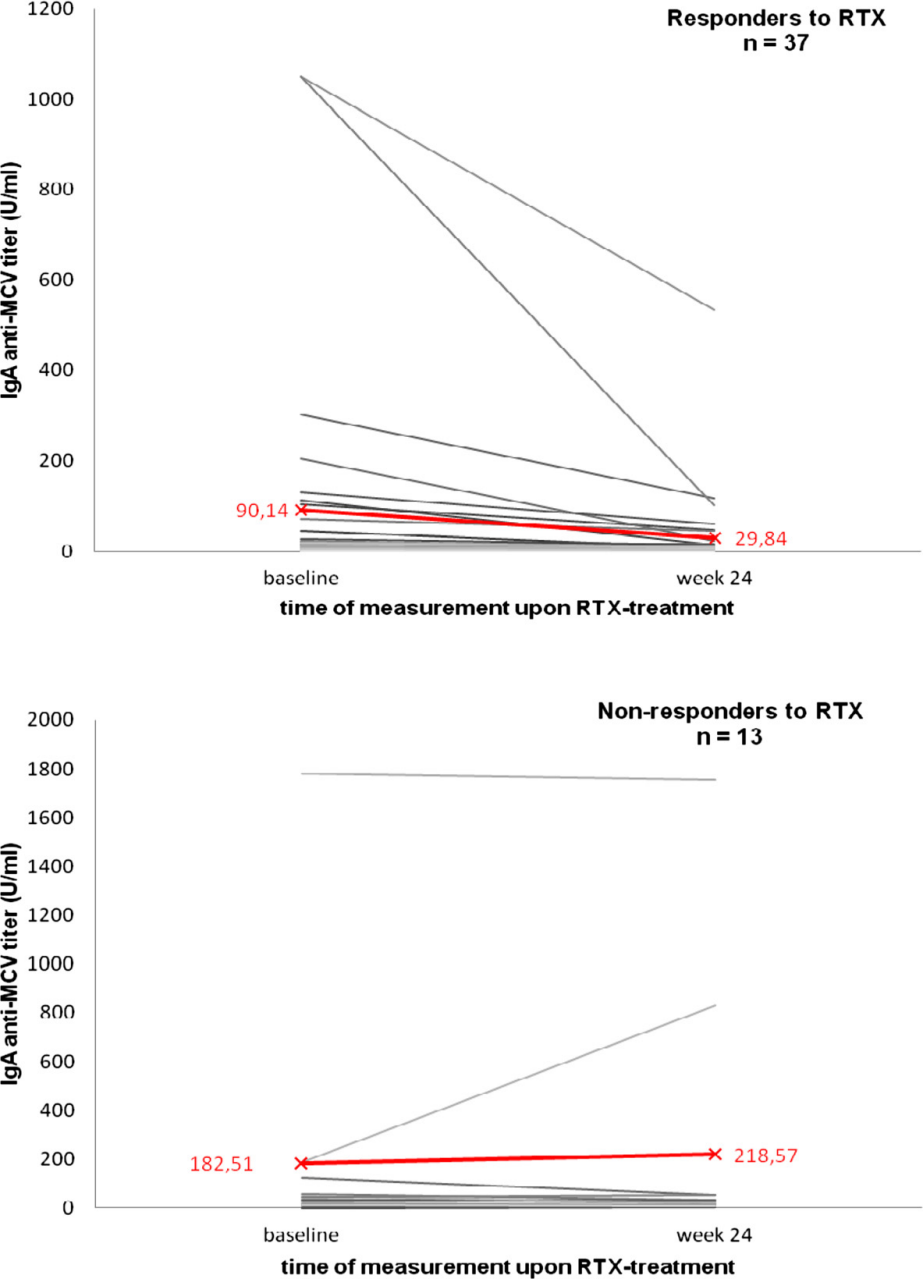
Individual IgA anti-MCV titer courses of responders and non-responders upon RTX treatment with mean titer change (red). Responders showed a greater tendency to reduce their initial titer from baseline to week 24 upon RTX treatment compared to non-responders, who rather remained stable

For quantitative analysis, we determined the mean titer courses of AMCV isotypes upon RTX treatment in RRs and NRRs and compared them with RF subtypes and ACCP IgG (Tables 1 and 2, Fig. 4). RRs showed lower mean titers of all AMCV subtypes at baseline, with a further substantial reduction upon treatment. In contrast, NRRs exhibited higher AMCV titers at baseline, which were less sensitive to change despite treatment (Fig. 4). Most notably, the mean baseline AMCV IgA titer of RRs decreased by nearly 67 % (from 90.14 to 29.84 U/ml; *P* <0.0001), whereas the titer of NRRs was twice as high at baseline and rather remained stable, with a non-significant increase of 19.76 % during the follow-up period (from 182.51 to 218.57; *P* = 0.17) (Fig. 5). To evaluate a clear difference of AMCV IgA variations between RRs and NRRs without bias of seronegative patients, we considered the titer changes of AMCV IgA-positive RRs and NRRs. The tendency became more obvious: a similar baseline titer in RRs and NRRs (261.55 versus 259.99 U/ml) decreased significantly in RRs by 69 % (to 81.56 U/ml; *P* = 0.0005), but increased slightly in NRRs by 20 % (to 312.25 U/ml; *P* = 0.2). As a result, the change of AMCV IgA in RRs was significantly different compared to NRRs at the end of the follow-up period (*P* = 0.007).

With respect to AMCV IgM, a titer decrease was observed in both RRs and NRRs, but with an increased decline in RRs (Table 1). In detail, AMCV IgM titers decreased significantly by 73.95 % in RRs (*P* <0.0001) and by 62.5 % in NRRs (*P* = 0.03), with a substantial difference between both groups at week 24 (*P* = 0.0003) (Fig. 4). More seropositive RRs normalized their titer levels for AMCV IgM and AMCV IgA under the cut-off level than NRRs after treatment. The number of patients who normalized their AMCV IgA levels was less frequent compared to AMCV IgM.

Such autoantibody kinetics were not observed for RF subtypes and ACCP IgG (Table 2). Although the mean baseline titers of RF IgG, IgM and IgA (with the exception of ACCP IgG in NRRs) decreased significantly with treatment in RRs and NRRs (*P* <0.0001), the difference did not reach statistical significance at week 24 (Mann–Whitney U *P* >0.05) (Fig. 4).

### IgA anti-mutated citrullinated vimentin titers in relation to Disease Activity Score 28 treatment response

**Fig. 6:**
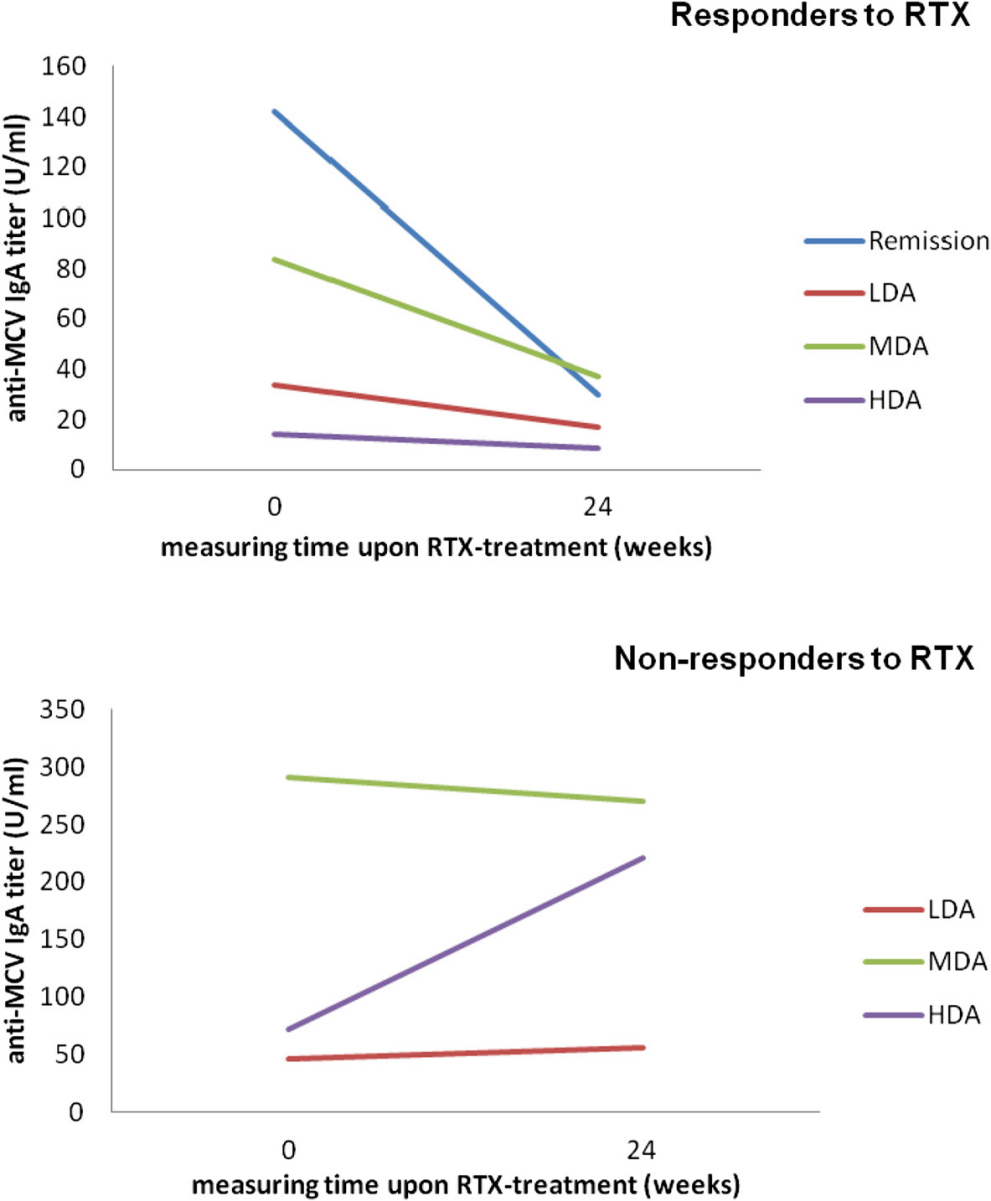
Mean IgA anti-MCV titer course for responders and non-responders to RTX corresponding to their achieved Disease Activity Score 28 (DAS28) after RTX treatment. The achieved DAS28 and treatment response at week 24 according to the EULAR response criteria were reflected by the extent of anti-MCV IgA titer reductions. The better the DAS28 responses were, the greater the IgA anti-MCV reductions were. Responders in remission showed the greatest IgA anti-MCV reductions, whereas non-responders with high disease activity slightly increased their initial levels. EULAR, European League against Rheumatism; HDA, high disease activity; LDA, low disease activity; MDA, moderate disease activity

AMCV IgA titer reductions reflected improved treatment response according to the DAS28 and the EULAR response criteria (Table 3). In detail, RRs who achieved remission at week 24 had the greatest percentage reductions in AMCV IgA titers, RRs with low and moderate disease activity presented moderate decreases and RRs with high disease activity revealed only a minor decline in AMCV IgA titers (Fig. 6). In comparison, NRRs with moderate disease activity at week 24 had rather unchanged AMCV IgA titers over time and NRRs with high disease activity even showed an increase of AMCV IgA titers (Fig. 6). Interestingly, the absolute titer exerted no influence. Thus, only the relative change of AMCV IgA titers from baseline to the end of the follow-up period provides information about the response characteristic.

**Table 3:** Anti-MCV IgA titer courses of responders and non-responders to RTX in relation to their achieved DAS28 response after six months of RTX treatment (according to the EULAR response criteria)

Responders to RTX (RRs)			
DAS28 response of 37 RRs at week 24 (number of RRs)	mean AMCV IgA titer at baseline (U/ml)	mean AMCV IgA titer at week 24 (U/ml)	percentage change (absolute titer change)
DAS28 ≤2.6 remission (12/37)	141.94	30.00	78.86 % (111.93)
DAS28 ≤3.2 and >2.6 low disease activity (5/37)	33.85	17.06	49.61 % (16.79)
DAS28 ≤5.1 and >3.2 moderate disease activity (17/37)	83.52	37.26	55.39 % (46.26)
DAS28 >5.1 high disease activity (3/37)	14.32	8.45	41.00 % (8.87)
Non-responders to RTX (NRRs)			
DAS28 response of 13 NRRs at week 24 (number of NRRs)	mean AMCV IgA titer at baseline (U/ml)	mean AMCV IgA titer at week 24 (U/ml)	percentage change (absolute titer change)
DAS28 ≤2.6 remission (0/13)	/	/	/
DAS28 ≤3.2 and>2.6 low disease activity (1/13)	46.11	55.56	17.01 % (9.45)
DAS28 ≤5.1 and >3.2 moderate disease activity (7/13)	290.79	270.51	06.97 % (20.27)
DAS28 >5.1 high disease activity (5/13)	71.41	221.49	67.76 % (150.08)

AMCV, antibodies against mutated citrullinated vimentin; DAS28, 28-joint Disease Activity Score; EULAR, European League against Rheumatism; NRRs, non-responders to RTX; RRs, responders to RTX; RTX, rituximab

**Fig. 7:**
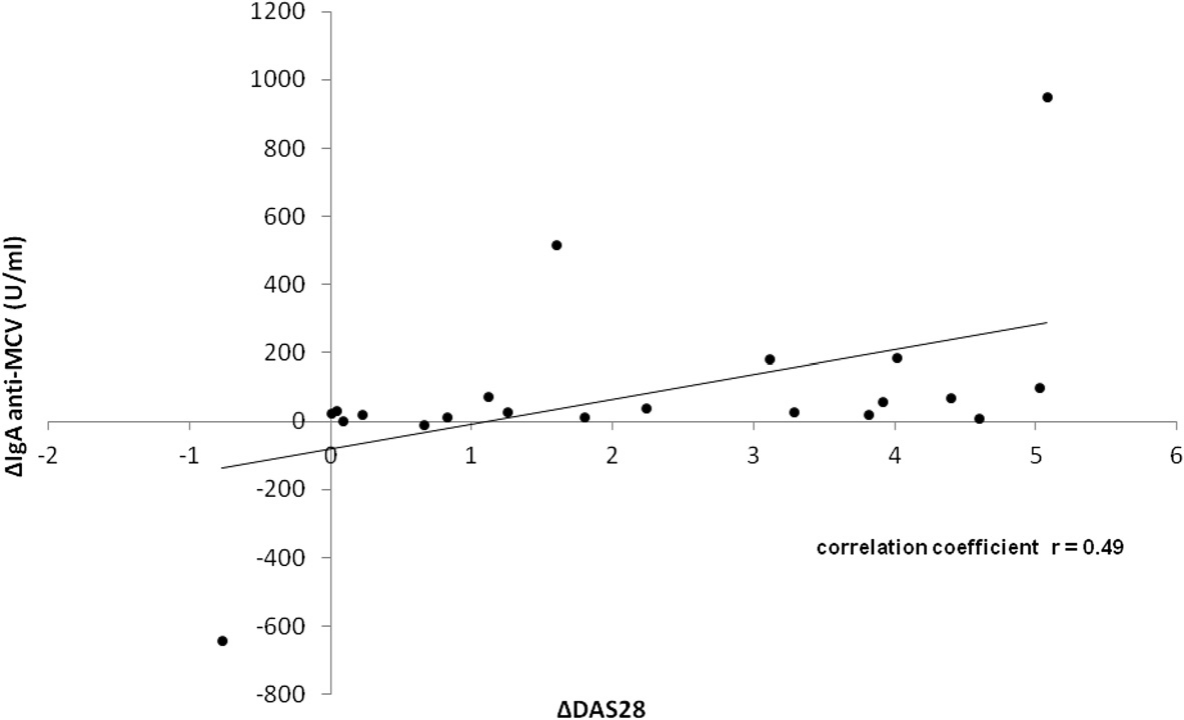
Correlation of IgA anti-MCV and Disease Activity Score 28 (DAS28) in IgA anti-MCV-positive patients. The difference (delta) of anti-MCV IgA titers (delta anti-MCV IgA) from baseline to 24 weeks after RTX treatment correlated moderately with a greater improvement of DAS28 (delta DAS28) in anti-MCV IgA-positive patients (n = 21) (r = 0.49)

In line with these findings, a moderate correlation of AMCV IgA and DAS28 could be described (Fig. 7). In order to determine a purely quantitative relation between AMCV IgA and DAS28 without bias of two different time points, we correlated the difference from baseline to the end of the follow-up period in titers of AMCV IgA-positive patients (n = 21) in relation to their change in DAS28. A correlation coefficient of r = 0.49 was found (*P* = 0.023). The greater the difference and reduction of AMCV IgA titers were, the greater the difference and improvement in DAS28 were. This was in agreement with the above-mentioned AMCV IgA reductions in relation to their DAS28 response. In contrast, no significant correlation was observed for the other autoantibody subtypes and DAS28 values (data not shown).

### Coincidence of autoantibody subtypes in IgA antibody against mutated citrullinated vimentin-positive patients

**Fig. 8:**
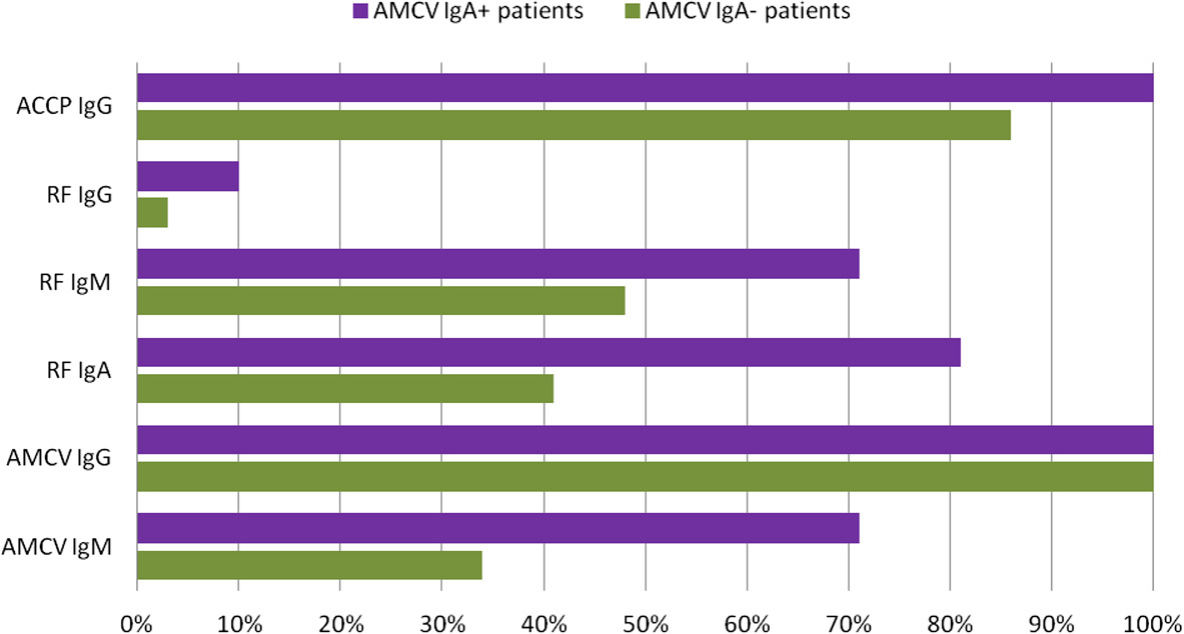
Coincidence of autoantibody subtypes with anti-MCV (AMCV) IgA-positive and -negative patients at baseline. AMCV IgA-positive patients (purple) showed a greater overlap with RA-associated autoantibodies than AMCV IgA-negative patients (green), with significant coincidence of AMCV IgA, IgM and RF IgA

Interestingly, in our AMCV IgG-positive cohort, almost all patients were also positive for ACCP IgG (n = 46 out of 50) but negative for RF IgG (n = 47 out of 50) (Tables 1 and 2). A separation into AMCV IgA-positive (n = 21) versus AMCV IgA-negative (n = 29) patients revealed a higher association of the other AMCV subtypes, with consequent higher mean titers of IgM and IgG in the AMCV IgA-positive group (Table 4). In detail, AMCV IgG titers were twofold and IgM titers threefold higher in the AMCV IgA-positive group compared to the negative group (*P* = 0.012). Furthermore, only the subtype IgA of RF showed a significant coincidence with AMCV IgA. ACCP IgG and RF IgM were not significantly associated with AMCV IgA (*P* <0.05) (Fig. 8).

**Table 4:** Coincidence of autoantibody subtypes with anti-MCV IgA (AMCV IgA)-positive and -negative patients respecting seropositivity and mean titers at baseline

		AMCV IgA-positive patients	AMCV IgA-negative patients	Mann–Whitney U *P* value
		(n = 21)	(n = 29)	
AMCV IgG	positive (%, n)	100 % (21/21)	100 % (29/29)	
	negative (%, n)	0	0	
	mean titer (U/ml)	1128.58	548.13	0.012
	SD (U/ml)	1160.25	573.98	
AMCV IgM	positive (%, n)	71 % (15/21)	34 % (10/29)	
	negative (%, n)	29 % (6/21)	66 % (19/29)	
	mean titer (U/ml)	98.65	27.1	0.005
	SD (U/ml)	157.69	47.63	
RF IgG	positive (%, n)	10 % (2/21)	3 % (1/29)	
	negative (%, n)	90 % (19/21)	97 % (28/29)	
	mean titer (IU/ml)	16.42	9.54	0.013
	SD (IU/ml)	15.62	14.13	
RF IgM	positive (%, n)	71 % (15/21)	48 % (14/29)	
	negative (%, n)	29 % (6/21)	52 % (15/29)	
	mean titer (IU/ml)	83.47	34.96	0.071
	SD (IU/ml)	103.29	51.77	
RF IgA	positive (%, n)	81 % (17/21)	41 % (12/29)	
	negative (%, n)	19 % (4/21)	59 % (17/29)	
	mean titer (IU/ml)	161.68	88.66	0.003
	SD (IU/ml)	137.91	120.54	
ACCP IgG	positive (%, n)	100 % (21/21)	86 % (25/29)	
	negative (%, n)	0 % (0/21)	14 % (4/29)	
	mean titer (U/ml)	1262.77	989.99	0.253
	SD (U/ml)	597.03	776.62	

ACCP, antibodies against cyclic citrullinated peptide; AMCV, antibodies against mutated citrullinated vimentin; n, number of patients; NRRs, non-responders to RTX; RF, rheumatoid factor; RRs, responders to RTX; RTX, rituximab; SD, standard deviation

## Discussion

The objective of this study was to identify a predictive biomarker among RA patients undergoing BCDT with RTX. Since seropositivity for RA-associated autoantibodies are accepted indicators for response to RTX, we investigated whether differences in fine reactivity and Ig isotype kinetics among AMCV-positive patients could provide additional information. Since the pathogenetic role of these immunoglobulin isotypes is also of interest, we compared the profile of AMCV antibodies to established RF subtypes and ACCP IgG.

Analysis of AMCV IgG-positive patients revealed differences with respect to MCV epitope recognition patterns, serological baseline status and follow-up changes in relation to response outcome. Patients with response to RTX were characterized by a restrictive antibody response of AMCV IgG, decreasing mean titers and reduction of initially recognized epitope pattern upon treatment. In contrast, double-seropositivity for AMCV IgG and IgA (and IgM, but to a lesser degree), stable antibody mean titers and an unchanged or even expanded epitope recognition pattern during the follow-up period were associated with non-response to RTX. These findings were confirmed by a moderate correlation of AMCV IgA reductions with improved DAS28 response.

In general, non-response to RTX could be due to resistant B-cell repertoire or/and pharmacokinetic differences in individual cases, like different tissue penetration of RTX to lymphoid niches [23]. In this context, the results of our study provide evidence for a RTX-sensitive B-cell subpopulation in RRs with a reduced epitope recognition pattern and decreasing IgG titers under treatment. In contrast, an unchanged or even expanded reactivity after B-cell depletion could be explained by active IgG-producing B-cell subsets in NRRs, which are less sensitive to RTX. Furthermore, a higher coincidence of AMCV isotypes, including IgA, in NRRs could be related to refractory IgA-producing B-cell subsets.

A close link of RTX response to CD20-positive memory B-cell compartment has been shown by previous studies. In most of these investigations, NRRs had an incomplete depletion and higher repopulation numbers of memory B-cells [24–26]. These memory B-cells are involved in the formation of secondary germinal centers and differentiate into CD20-negative plasmablasts and plasma cells, contributing to active inflammatory responses with rise in antibody levels [27]. Both isotypes IgA and IgG are mainly produced by tissue-resident memory B-cells and plasma cells [28, 29]. Since the distribution and architecture of these compartments can vary between individuals, RTX can cause distinct depletion of these B-cells with individually varying IgA or IgG kinetics [30]. In this context, stable AMCV IgA titers in both RRs and NRRs could be explained by lower sensitivity of AMCV IgA-producing B-cells towards RTX treatment. As shown also by vaccination studies, BCDT affects mainly short-lived CD20-positive B-cells, explaining especially the decline of IgM titers, which was also observed in our cohort [31].

The association of non-response to RTX with the appearance of IgA anti-MCV points to the significance of this Ig subset in RA. At early stages of RA investigations, the presence of IgA RF was shown to be associated with increased disease activity and severity [32]. Furthermore, IgA RF also preceded disease onset and was linked to extraarticular manifestations and resistance to treatment with TNFi [33, 34]. Of note, overall serum IgA and IgG levels have been described as rather stable, even after repeated cycles of RTX in RA patients [35]. Finally, co-expression of IgA and IgG for AMCV has been associated with disease severity by another prior study [19], which supports the relevance of an interaction between the mucosal and systemic humoral responses. Mei et al. have identified the source of serum IgA from circulating plasmablasts and suggested their origin to be from mucosal memory B-cells [36]. Due to loss of CD20 receptors, these mucosal plasmablasts were not depleted by RTX [37], and subsequently, levels of IgA remained stable. As known from vaccination studies, IgA responses were mainly induced by oral but not systemic exposure to antigen within the mucosa-associated lymphoid tissues [38]. Thus, the appearance of IgA autoantibodies suggests primarily mucosal triggers for such autoimmune responses. This connection of mucosal immune reactions and autoimmune disorders is also supported by known oral or gastrointestinal triggers in RA [39, 40], including nutrition-dependent increase in antibody titers [41]. In summary, it can be hypothesized that tissue-resident memory B-cells could represent the main source for such plasmablasts, causing treatment resistance to RTX.

Our study is limited by the relatively small sample size and inclusion of patients from two cohorts, of whom one part received RTX as a first-line biologic. Thus, different pretreatment with biologicals and concomitant medication could have influenced our results. Furthermore, by preselecting for AMCV IgG-positive patients, we could have missed different subtype kinetics in other serologic subgroups. Although this study design limits the comparability to existing studies, we were able to perform detailed analyses of the autoantibody kinetics in RTX-treated patients. In summary, our results confirm a correlation of AMCV subtypes with disease activity, and provide evidence for their potential prediction and monitoring value.

## Conclusions

Restrictive baseline seropositivity for anti-MCV IgG was identified as a predictor for response to RTX. Co-expression of IgA was associated with treatment failure to RTX. Reduction of AMCV IgA titers under RTX showed a correlation with EULAR response in RA. Furthermore, rigid IgA isotype under such targeted therapies could be related to refractory B-cell subsets, giving evidence of greater heterogeneity within the so called seropositive RA cohort.

## Additional files

Additional file 1:
**Patients’ characteristics.** (DOCX 20 kb) Additional file 2: Table S2. Sequence of selected mutated citrullinated vimentin (MCV) with epitopes from 1 to 88. The entire protein sequence of MCV was cut into 88 peptides consisting of 17 amino acids, with 12 overlapping amino acids to the neighbourhood peptide, with following amino acid code: X = citrulline; X = mutated glycine = citrulline; H = mutated serine = histidine. (DOCX 18 kb) Additional file 3: Figure S1. ROC curve analysis for AMCV IgA assay. Cut-off level at 20 U/ml resulted in a specificity of 100 % and sensitivity of 42 %. (DOCX 23 kb)

## Abbreviations

ACCP: antibodies against cyclic citrullinated peptide/protein
ACPA: anti-cyclic citrullinated protein antibodies
AMCV: antibodies against mutated citrullinated vimentin
BCDT: B-cell depletion therapy
DAS28: Disease Activity Score in 28 joints
DMARDs: disease-modifying anti-rheumatic drugs
ESR: erythrocyte sedimentation rate
EULAR: European League against Rheumatism
HDA: high disease activity
IgG/ IgM/ IgA: Immunoglobulin G/ Immunoglobulin M/ Immunoglobulin A
LDA: low disease activity
MDA: moderate disease activity
MTX: methotrexate
NRRs: non-responders to RTX
RA: rheumatoid arthritis
RF: rheumatoid factor
ROC: receiver operating curve
RRs: responders to RTX
RTX: rituximab
SD: standard deviation
TNFi: Tumor necrosis factor- alpha inhibitor
