# Supplementary material for: Restrictive IgG antibody response against mutated citrullinated vimentin predicts response to rituximab in patients with rheumatoid arthritis
Source: Arthritis Res Ther. 2015 Aug 13;17(1):206. doi: 10.1186/s13075-015-0717-z (PMC4535682; doi:10.1186/s13075-015-0717-z)
Supplement: Additional file 1: — Patients’ characteristics. (DOCX 20 kb) [file 13075_2015_717_MOESM1_ESM.docx]

Additional file 1: Patients´ characteristics
