# Supplementary material for: Restrictive IgG antibody response against mutated citrullinated vimentin predicts response to rituximab in patients with rheumatoid arthritis
Source: Arthritis Res Ther. 2015 Aug 13;17(1):206. doi: 10.1186/s13075-015-0717-z (PMC4535682; doi:10.1186/s13075-015-0717-z)
Supplement: Additional file 2: Table S2. — Sequence of selected mutated citrullinated vimentin (MCV) with epitopes from 1 to 88. The entire protein sequence of MCV was cut into 88 peptides consisting of 17 amino acids, with 12 overlapping amino acids to the neighbourhood peptide, with following amino acid code: X = citrulline; X = mutated glycine = citrulline; H = mutated serine = histidine. (DOCX 18 kb) [file 13075_2015_717_MOESM2_ESM.docx]

**Additional file 2**

**Table 2: Sequence of selected citrullinated-mutated vimentin with epitopes from 1 to 88. The entire protein sequence of MCV was cut into 88 peptides consisting of 17 amino acids with 12 overlapping amino acids to the neighbourhood peptide with following amino acid code:**

**X = citrulline; X = mutated glycine = citrulline; H = mutated serine = histidine.**

| peptide number | Sequence of citrullinated-mutated peptides | peptide number | Sequence of citrullinated-mutated peptides |
| --- | --- | --- | --- |
| 1 | ST**X**SVSSSSY**XX**MFGXP-NH2 | 45 | **X**KVESLQEEIAFLKKLH-NH2 |
| 2 | SSSSY**XX**MFGXPGTAS**X**-NH2 | 46 | LQEEIAFLKKLHEEEIQ-NH2 |
| 3 | **XX**MFGXPGTAS**X**PSSS**X**-NH2 | 47 | AFLKKLHEEEIQELQAQ-NH2 |
| 4 | GPGTAS**X**PSSS**X**SYVTT-NH2 | 48 | LHEEEIQELQAQIQEQH-NH2 |
| 5 | S**X**PSSS**X**SYVTTST**X**TY-NH2 | 49 | IQELQAQIQEQHVQIDV-NH2 |
| 6 | S**X**SYVTTST**X**TYSLGSA-NH2 | 50 | AQIQEQHVQIDVDVSKP-NH2 |
| 7 | TTST**X**TYSLGSAL**X**PST-NH2 | 51 | QHVQIDVDVSKPDLTAA-NH2 |
| 8 | TYSLGSAL**X**PSTS**X**HLY-NH2 | 52 | DVDVSKPDLTAAL**X**DV**X**-NH2 |
| 9 | SAL**X**PSTS**X**HLYASSPG-NH2 | 53 | KPDLTAAL**X**DV**X**QQYES-NH2 |
| 10 | STS**X**HLYASSPGXVYAT-NH2 | 54 | AAL**X**DV**X**QQYESVAAKN-NH2 |
| 11 | LYASSPGXVYAT**X**SSAV-NH2 | 55 | V**X**QQYESVAAKNLQEAE-NH2 |
| 12 | PGXVYAT**X**SSAV**X**L**X**SS-NH2 | 56 | ESVAAKNLQEAEEWYKS-NH2 |
| 13 | AT**X**SSAV**X**L**X**SSVPGV**X**-NH2 | 57 | KNLQEAEEWYKSKFADL-NH2 |
| 14 | AV**X**L**X**SSVPGV**X**LLQDS-NH2 | 58 | AEEWYKSKFADLSEAAN-NH2 |
| 15 | SSVPGV**X**LLQDSVDFSL-NH2 | 59 | DLSEAAN**X**NNDAL**X**QAK-NH2 |
| 16 | V**X**LLQDSVDFSLADAIN-NH2 | 60 | KSKFADLSEAAN**X**NNDA-NH2 |
| 17 | DSVDFSLADAINTEFKN-NH2 | 61 | AN**X**NNDAL**X**QAKQESTE-NH2 |
| 18 | SLADAINTEFKNT**X**TNE-NH2 | 62 | DAL**X**QAKQESTEY**XX**QV-NH2 |
| 19 | KNT**X**TNEKVELQELND**X**-NH2 | 63 | AKQESTEY**XX**QVQSLTC-NH2 |
| 20 | INTEFKNT**X**TNEKVELQ-NH2 | 64 | TEY**XX**QVQSLTCEVDAL-NH2 |
| 21 | NEKVELQELND**X**FANYI-NH2 | 65 | QVQSLTCEVDALKGTNE-NH2 |
| 22 | LQELND**X**FANYIDKV**X**F-NH2 | 66 | TCEVDALKGTNESLE**X**Q-NH2 |
| 23 | D**X**FANYIDKV**X**FLEQQN-NH2 | 67 | ALKGTNESLE**X**QM**X**EME-NH2 |
| 24 | YIDKV**X**FLEQQNKILLA-NH2 | 68 | NESLE**X**QM**X**EMEENFAV-NH2 |
| 25 | **X**FLEQQNKILLAELEQL-NH2 | 69 | **X**QM**X**EMEENFAVEAANY-NH2 |
| 26 | QNKILLAELEQLKGQGK-NH2 | 70 | IG**X**LQDEIQNMKEEMA**X**-NH2 |
| 27 | GKS**X**LGDLYEEEM**X**EL**X**-NH2 | 71 | AVEAANYQDTIG**X**LQDE-NH2 |
| 28 | QLKGQGKS**X**LGDLYEEE-NH2 | 72 | NYQDTIG**X**LQDEIQNMK-NH2 |
| 29 | LAELEQLKGQGKS**X**LGD-NH2 | 73 | MEENFAVEAANYQDTIG-NH2 |
| 30 | GDLYEEEM**X**EL**XX**QVDQ-NH2 | 74 | DEIQNMKEEMA**X**HL**X**EY-NH2 |
| 31 | EEM**X**EL**XX**QVDQLTNDK-NH2 | 75 | MKEEMA**X**HL**X**EYQDLLN-NH2 |
| 32 | L**XX**QVDQLTNDKA**X**VEV-NH2 | 76 | A**X**HL**X**EYQDLLNVKMAL-NH2 |
| 33 | DQLTNDKA**X**VEVE**X**DNL-NH2 | 77 | EYQDLLNVKMALDIEIA-NH2 |
| 34 | DKA**X**VEVE**X**DNLAEDIM-NH2 | 78 | LNVKMALDIEIATY**X**KL-NH2 |
| 35 | EVE**X**DNLAEDIM**X**L**X**EK-NH2 | 79 | ALDIEIATY**X**KLLEGEE-NH2 |
| 36 | NLAEDIM**X**L**X**EKLQEEM-NH2 | 80 | IATY**X**KLLEGEES**X**ISL-NH2 |
| 37 | IM**X**L**X**EKLQEEMLQ**X**EE-NH2 | 81 | KLLEGEES**X**ISLPLPNF-NH2 |
| 38 | EKLQEEMLQ**X**EEAENTL-NH2 | 82 | EES**X**ISLPLPNFSSLNL-NH2 |
| 39 | EMLQ**X**EEAENTLQSF**X**Q-NH2 | 83 | SLPLPNFSSLNL**X**ETNL-NH2 |
| 40 | EEAENTLQSF**X**QDVDNA-NH2 | 84 | NL**X**ETNLDSLPLVDTHS-NH2 |
| 41 | TLQSF**X**QDVDNASLA**X**L-NH2 | 85 | NFSSLNL**X**ETNLDSLPL-NH2 |
| 42 | **X**QDVDNASLA**X**LDLE**X**K-NH2 | 86 | NLDSLPLVDTHSK**X**TFL-NH2 |
| 43 | **X**LDLE**X**KVESLQEEIAF-NH2 | 87 | PLVDTHSK**X**TFLIKTVE-NH2 |
| 44 | NASLA**X**LDLE**X**KVESLQ-NH2 | 88 | HSK**X**TFLIKTVET**X**DGQ-NH2 |
