# Supplementary material for: Restrictive IgG antibody response against mutated citrullinated vimentin predicts response to rituximab in patients with rheumatoid arthritis
Source: Arthritis Res Ther. 2015 Aug 13;17(1):206. doi: 10.1186/s13075-015-0717-z (PMC4535682; doi:10.1186/s13075-015-0717-z)

Supplementary material

Figure 1: ROC curve analysis for AMCV IgA assay. Cut-off at 20 U/ml resulted in a specificity of 100% and sensitivity of 42%. (TPF true positive fraction; FPF false positive fraction; AUROC Area under the Receiver Operating Characteristic)

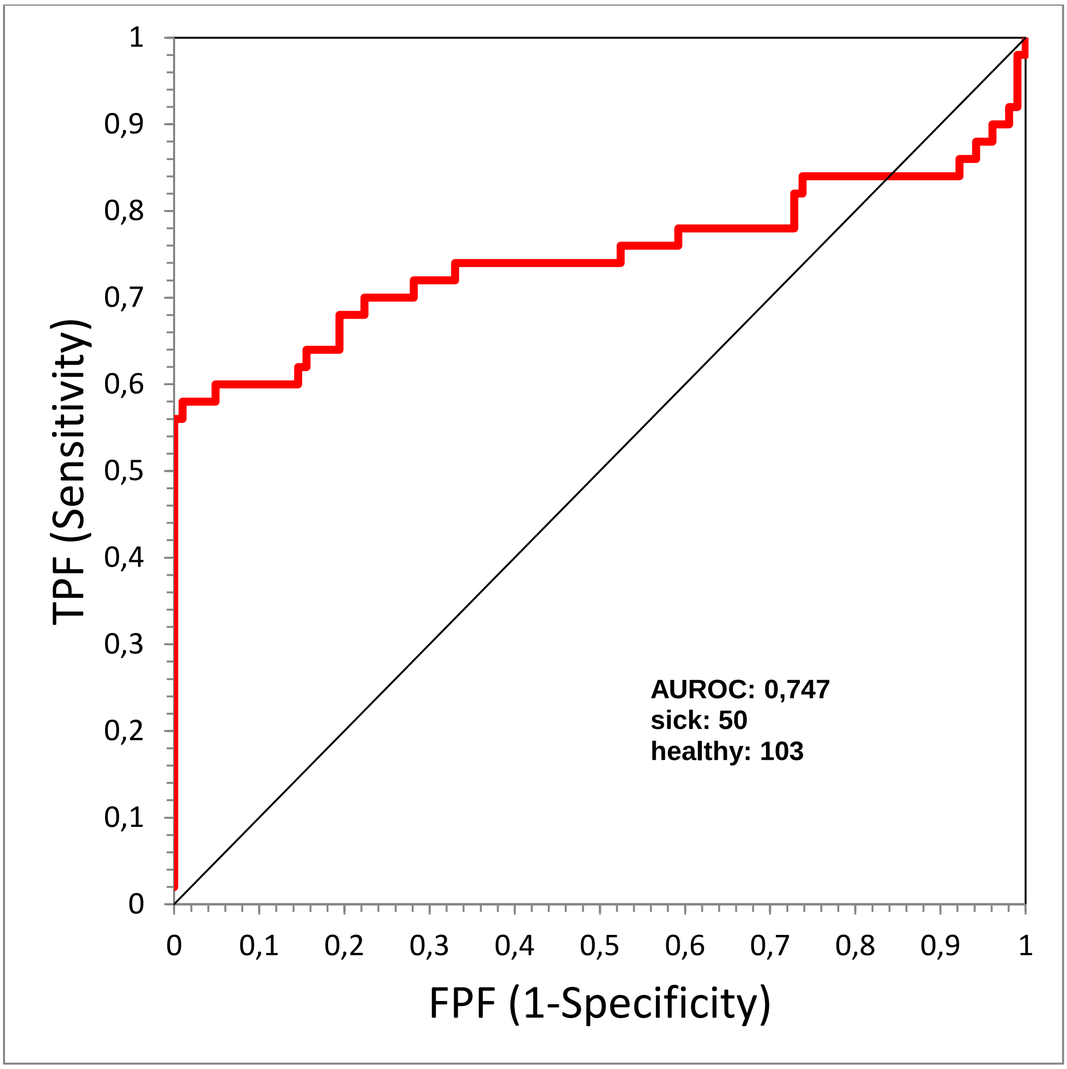

Supplement: Additional file 3: Figure S1. — ROC curve analysis for AMCV IgA assay. Cut-off level at 20 U/ml resulted in a specificity of 100 % and sensitivity of 42 %. (DOCX 23 kb) [file 13075_2015_717_MOESM3_ESM.pdf]
